# Supplementary material for: New insights in the coordinated amidase and glucosaminidase activity of the major autolysin (Atl) in Staphylococcus aureus
Source: Commun Biol. 2020 Nov 20;3:695. doi: 10.1038/s42003-020-01405-2 (PMC7679415; doi:10.1038/s42003-020-01405-2)
Supplement: Supplementary file 1 — Supplementary Information [file 42003_2020_1405_MOESM1_ESM.pdf]

Supplementary Materials

New insights in the coordinated amidase and glucosaminidase activity of the major autolysin (Atl) in *Staphylococcus aureus*

Mulugeta Nega[1], Paula Maria Tribelli[1,2], Katharina Hipp[3], Mark Stahl[4], and Friedrich Götz[1*]

Supplementary Fig. 1. Increasing enlargments of TEM images of WT and mutants.

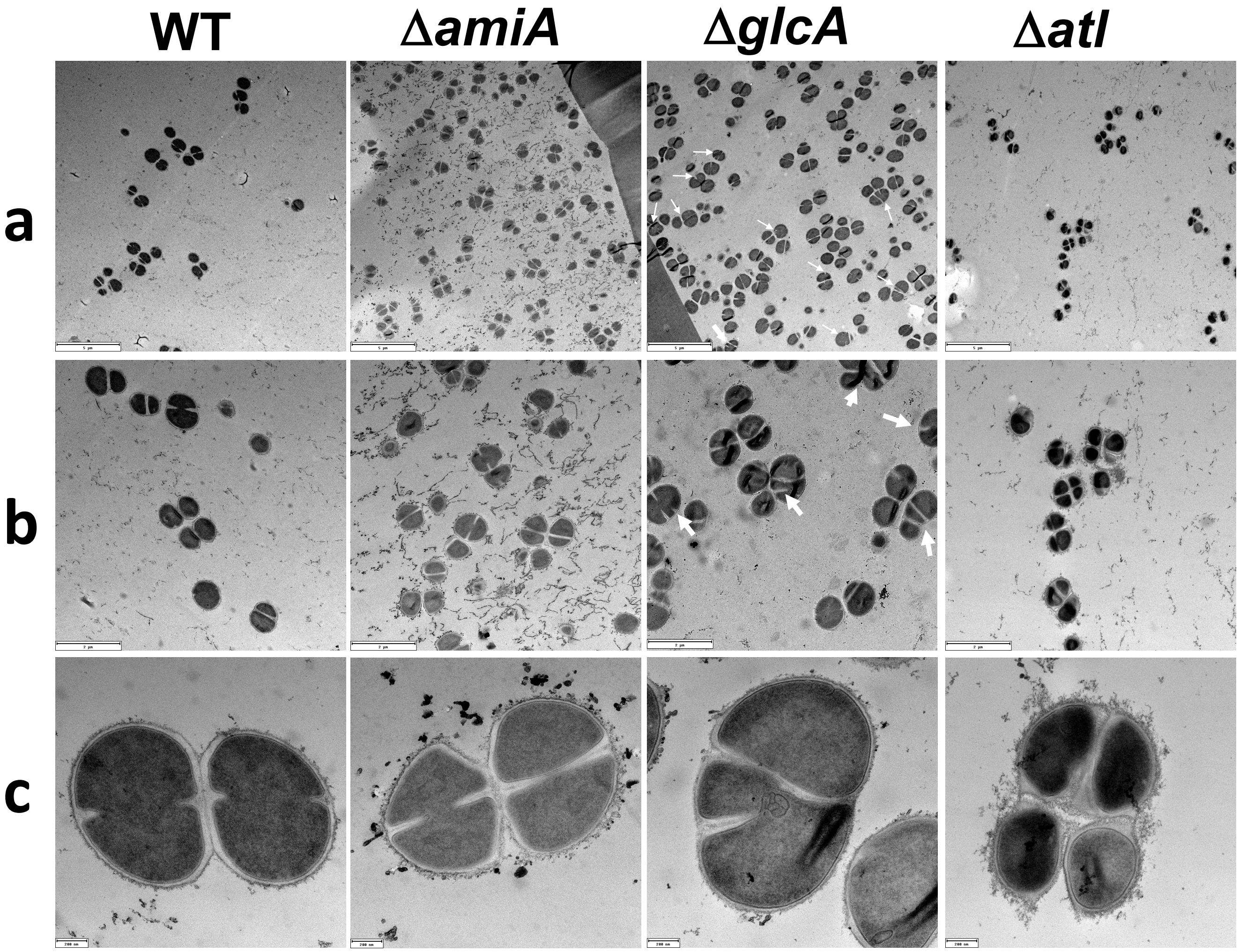

WT cells show proper septum formation, cell division and separation. In ΔamiA there is an accumulation of extracellular thread-like structures which represent presumably unprocessed PG. In ΔglcA, an asymmetric cell division is seen in many of the dividing cells. Finally, in the Δatl mutant the CW shows the most rigid structure of all mutants. Lots of extracellular thread-like PG structures can be seen and the daughter cells are tightly connected by unprocessed CW; however, no asymmetric cell division was observed. Enlargement scale bars: a) 5 µm; b) 2 µm; c) 200 nm

Supplementary Fig. 2. Relative amount of cells defect in proper septum formation.

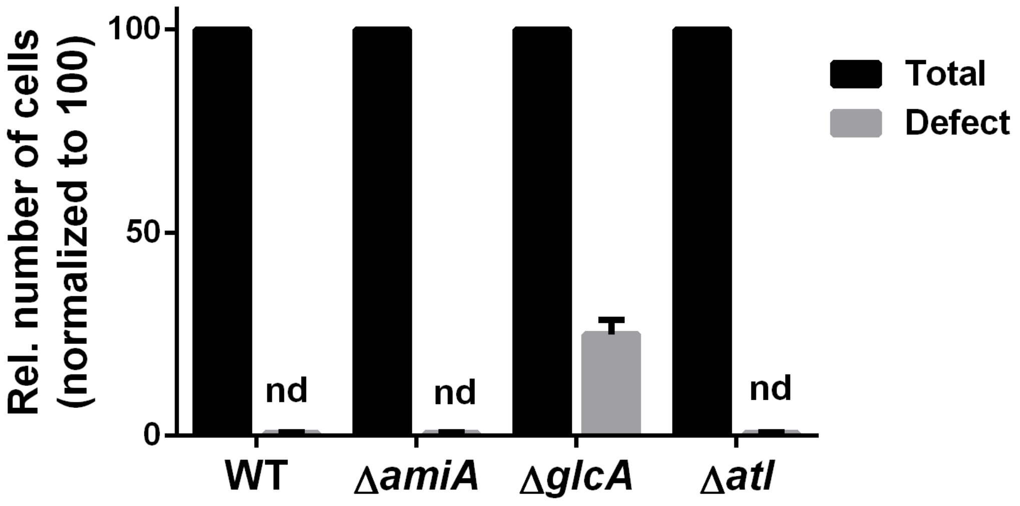

Quantitative Analysis of TEM images of SA113 WT, SA113Δ*amiA*, SA113Δ*glcA* and SA113Δ*atl* showed that about 20-30% of the cells of SA113Δ*glcA* have defect in proper septum formation and cell division (**nd:** *not detected*).

Supplementary Fig. 3. Comparative muropeptide RP-HPLC profiles after mutanolysin digestion of the WT and mutants PG.

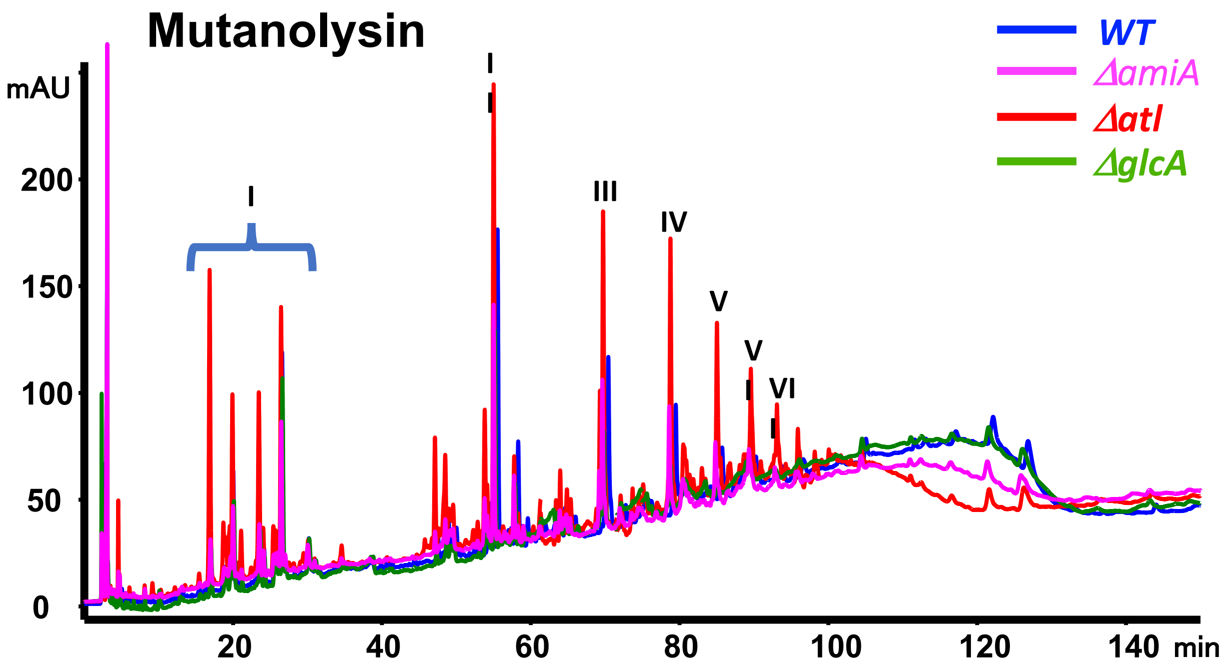

Comparison of the degree of crosslinking of the WT with mutants reveals that deletion of GlcA does not alter the crosslinking significantly while deletion of the AmiA leads to a ca. 30% reduction in PG crosslinking. As shown in Fig. 6, deletion of the whole AtlA leads to a 50% reduction in crosslinking.

Supplementary Fig. 4. Relative cluster size distribution of WT and mutants.

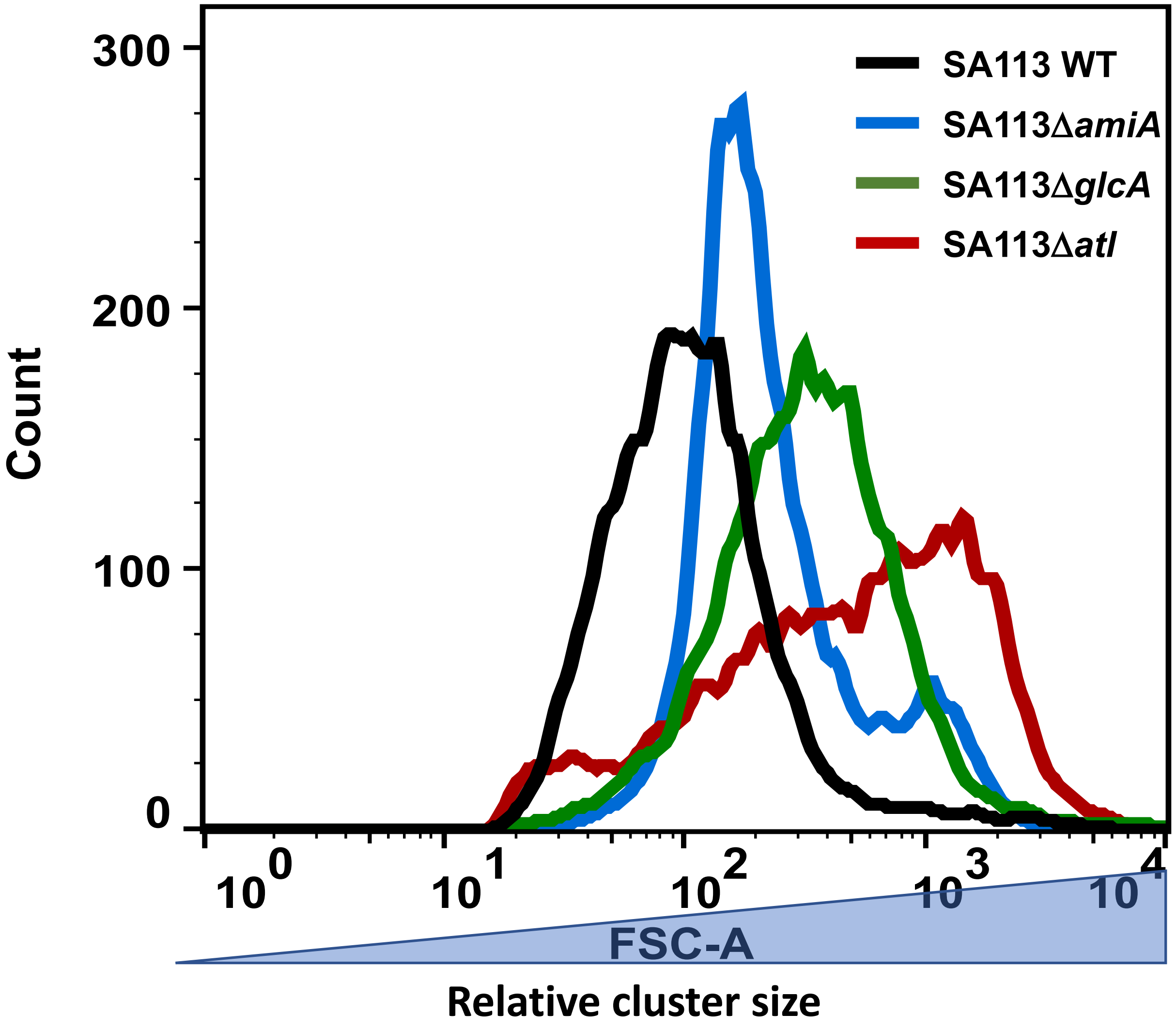

The relative cluster size distribution of the WT and deletion mutants were determined by forward scatter (FSC-A) flow cytometry of cells grown to mid exponential phase in TSB. SA113 WT (black), SA113Δ*amiA* (blue), SA113Δ*glcA* (green) and SA113Δ*atl* (red). Figure is representative of three independent experiments.

Supplementary Figure 5. Example of Flow Cytometry Gating

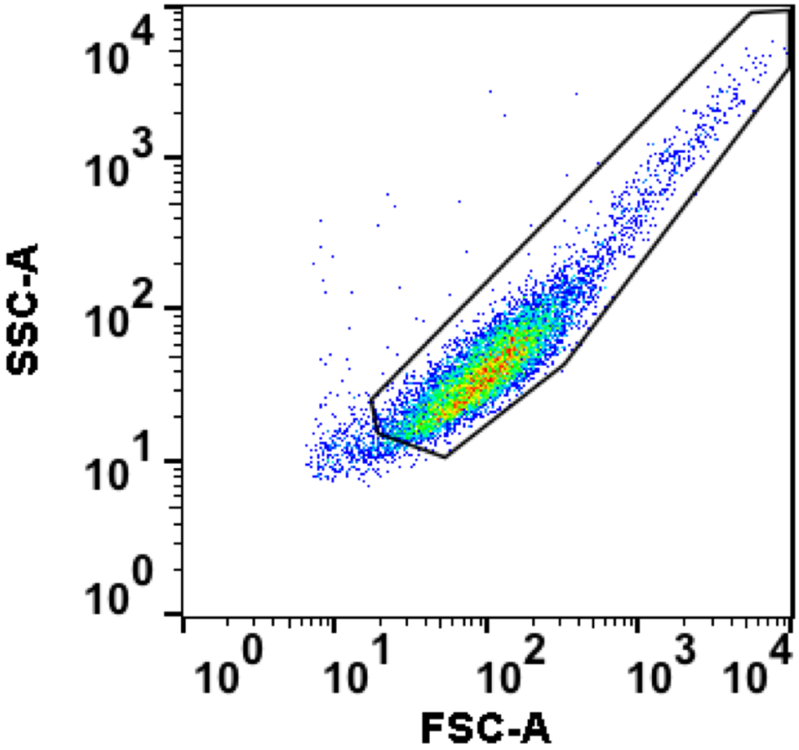

Prototypical example of two-dimensional Gaussian fit used for determination of gating from the SA113 WT sample

Supplementary Table 1. Primers used in this study

Name	Sequence
Glc_Up_Fwd	CGCGCAGATCTGTCGACGATATCGATCAATTATATGACTTAATTAAT GAAAAATATTTAATAAAAATG
Glc_Up_Rev	TTCATGTTGCTTATTATGCAGCAGGTAC
Glc_Down_Fwd	TAC CTGCTGCATAATAAGCAACATGAACATAGGA
Glc_Down_Rev	TGCAGGCATGCAAGCTTGATATCAACGTTTTGAAA TTTTTTCTAAATAG
Ami_Up_Fwd	CGCGCAGATCTGTCGACGATATCAAGAAAGAAAGT GTTTGTAGAAG
Ami_Up_Rev	CTTTTTTAGGACTAAAAGTAGTTACTTTTGGTG
Ami_Down_Fwd	TACTTTTAGTCCTAAAAAAGCTGTAGCAC
Ami_Down_Rev	TGCAGGCATGCAAGCTTGATATCACCTTTTAAGAATTGATTAATTTT ATCAATAG
GlcA_KO Fwd	AAGAAGCTGGTGCAGTTTCTGG
GlcA_KO Rev	GCAAAAGTAGCGGTGATTG
AmiA_KO Fwd	GCTGCTAAACCTGCTGCTCTTG
AmiA_KO Rev	CCGCATATTTTGCACCGTTT
pBASE6_Fwd	GGGCACCAATAACTGCCTTA
pBASE6_Rev	CGTCTTGCCTAACAACTACT

Supplementary Table 2. Amino acid composition and mass analysis of AmiA released peptide fragments of *S. aureus* PG

**Peptide**	**m/z calc.**	**m/z Obs.**
$AQK(G)_5AA$	772.818	773,440
$AQK(G)_5$ $[AQ\ (G)_5KA]AA$	1456,542	1456,819
$AQK(G)_5$ $[AQ\ (G)_5KA]_2AA$	2140,266	2140,299
$AQK(G)_5$ $[AQ\ (G)_5KA]_3AA$	2823,990	2823,861
$AQK(G)_5$ $[AQ\ (G)_5KA]_4AA$	3507,714	3507,236
$AQK(G)_5$ $[AQ\ (G)_5KA]_5AA$	4190,062	4190,753
$AQK(G)_5$ $[AQ\ (G)_5KA]_6AA$	4874,400	4874,270
$AQK(G)_5$ $[AQ\ (G)_5KA]_7AA$	5557,735	5557,631
$AQK(G)_5$ $[AQ\ (G)_5KA]_8AA$	6241,070	6241,171
$AQK(G)_5$ $[AQ\ (G)_5KA]_9AA$	6924,405	6924,679
$AQK(G)_5$ $[AQ\ (G)_5KA]_{10}AA$	7608,744	7609,011
$AQK(G)_5$ $[AQ\ (G)_5KA]_{11}AA$	8291,075	8290,972
$AQK(G)_5$ $[AQ\ (G)_5KA]_{12}AA$	8975,414	8975,952
