# Supplementary material for: New insights in the coordinated amidase and glucosaminidase activity of the major autolysin (Atl) in Staphylococcus aureus
Source: Commun Biol. 2020 Nov 20;3:695. doi: 10.1038/s42003-020-01405-2 (PMC7679415; doi:10.1038/s42003-020-01405-2)
Supplement: Supplementary file 3 — Description of Additional Supplementary Files [file 42003_2020_1405_MOESM3_ESM.pdf]

Description of Additional Supplementary Files

File Name: Supplementary Data

Description: Source data for figures 3, 6 and 10. Flow cytometry source data with percentage of gated cells (orange), mean FSC-A values (green) and total number of gated and counted cells (yellow) for Figure 3; Determination of the distribution of the monomeric and multimeric PG fragments in the WT, △atl and the plasmid complemented △atl PG by the relative areas of the corresponding HPLC chromatogram peaks for Fig 6 and RP-HPLC peak area units of MurNAc-GlcNAc and O-acetylated MurNAc-GlcNAc released by AmiA and GlcA digestion at different time points of 0 to 8 hours for Fig 10.
