# Supplementary material for: New insights in the coordinated amidase and glucosaminidase activity of the major autolysin (Atl) in Staphylococcus aureus
Source: Commun Biol. 2020 Nov 20;3:695. doi: 10.1038/s42003-020-01405-2 (PMC7679415; doi:10.1038/s42003-020-01405-2)
Supplement: Supplementary file 5 — Reporting Summary [file 42003_2020_1405_MOESM5_ESM.pdf]

nature research

Corresponding author(s): COMMSBIO-20-0733-T

Last updated by author(s): 2020.09.24

Reporting Summary

Nature Research wishes to improve the reproducibility of the work that we publish. This form provides structure for consistency and transparency in reporting. For further information on Nature Research policies, see Authors & Referees and the Editorial Policy Checklist .

Statistics

For all statistical analyses, confirm that the following items are present in the figure legend, table legend, main text, or Methods section.

n/a	Confirmed	
x		The exact sample size (*n*) for each experimental group/condition, given as a discrete number and unit of measurement
	x	A statement on whether measurements were taken from distinct samples or whether the same sample was measured repeatedly
x		The statistical test(s) used AND whether they are one- or two-sided<br>*Only common tests should be described solely by name; describe more complex techniques in the Methods section.*
x		A description of all covariates tested
x		A description of any assumptions or corrections, such as tests of normality and adjustment for multiple comparisons
x		A full description of the statistical parameters including central tendency (e.g. means) or other basic estimates (e.g. regression coefficient) AND variation (e.g. standard deviation) or associated estimates of uncertainty (e.g. confidence intervals)
x		For null hypothesis testing, the test statistic (e.g. *F*, *t*, *r*) with confidence intervals, effect sizes, degrees of freedom and *P* value noted<br>*Give P values as exact values whenever suitable.*
x		For Bayesian analysis, information on the choice of priors and Markov chain Monte Carlo settings
x		For hierarchical and complex designs, identification of the appropriate level for tests and full reporting of outcomes
x		Estimates of effect sizes (e.g. Cohen's *d*, Pearson's *r*), indicating how they were calculated

Our web collection on statistics for biologists contains articles on many of the points above.

Software and code

Policy information about availability of computer code

Data collection	HPLC data is collected by Agilent technologies, Chemstation for LC 3D systems, Ver. B.03.01 (317); HPLC/MS and Microscopy softwares described in the method description
Data analysis	HPLC data is analysed by Agilent technologies, Chemstation for LC 3D systems, Ver. B.03.01 (317); HPLC/MS and Microscopy softwares described in the method description. ChemDraw is used for chemical structure drawing and theoretical mass calculation.

For manuscripts utilizing custom algorithms or software that are central to the research but not yet described in published literature, software must be made available to editors/reviewers. We strongly encourage code deposition in a community repository (e.g. GitHub). See the Nature Research guidelines for submitting code & software for further information.

Data

Policy information about availability of data

All manuscripts must include a data availability statement. This statement should provide the following information, where applicable:

- Accession codes, unique identifiers, or web links for publicly available datasets
- A list of figures that have associated raw data
- A description of any restrictions on data availability

n/a

Field-specific reporting

Please select the one below that is the best fit for your research. If you are not sure, read the appropriate sections before making your selection.

[x] Life sciences   [ ] Behavioural & social sciences   [ ] Ecological, evolutionary & environmental sciences

Life sciences study design

All studies must disclose on these points even when the disclosure is negative.

Sample size	Figure 3, 6 and 10D are representative of three independent experiments.
Data exclusions	No data exclusions
Replication	Chromatographic, electron microscopic and flow cytometric analyses were repeated and replications were successful.
Randomization	No randomization was necessary
Blinding	No blindig necessary

Reporting for specific materials, systems and methods

We require information from authors about some types of materials, experimental systems and methods used in many studies. Here, indicate whether each material, system or method listed is relevant to your study. If you are not sure if a list item applies to your research, read the appropriate section before selecting a response.

Materials & experimental systems

n/a	Involved in the study
☒	☐ Antibodies
☒	☐ Eukaryotic cell lines
☒	☐ Palaeontology
☒	☐ Animals and other organisms
☒	☐ Human research participants
☒	☐ Clinical data

Methods

n/a	Involved in the study
☒	☐ ChIP-seq
☐	☒ Flow cytometry
☒	☐ MRI-based neuroimaging

Flow Cytometry

Plots

Confirm that:

☒ The axis labels state the marker and fluorochrome used (e.g. CD4-FITC).

☒ The axis scales are clearly visible. Include numbers along axes only for bottom left plot of group (a 'group' is an analysis of identical markers).

☒ All plots are contour plots with outliers or pseudocolor plots.

☒ A numerical value for number of cells or percentage (with statistics) is provided.

Methodology

Sample preparation	Sample was prepared as described in the method from an exponentially growing culture. No sample labelling was necessary.
Instrument	BD FACS Fortessa
Software	Data acquistion and processing was done by FlowJo software
Cell population abundance	There was only one post-sort fraction determined in the SSC/FSC plot that consisted the gated bacteria which was >90% of all measured events. Bacteria culture was diluted in sterile medium and media background was excluded from the set gate.
Gating strategy	Specific gating in the SSC/FSC plot was used to identify bacterial cell clustering and granulity.

☒ Tick this box to confirm that a figure exemplifying the gating strategy is provided in the Supplementary Information.
